# Standard work tools for managing pediatric baclofen pump infections and withdrawal

**DOI:** 10.1007/s00381-026-07219-7

**Published:** 2026-03-21

**Authors:** Rishi Jain, Benjamin E. Weiss, Elizabeth Snider, James M. Mossner, Jeffrey S. Raskin

**Affiliations:** 1 Division of Pediatric Neurosurgery, Ann and Robert H. Lurie Children’s Hospital, Chicago, IL USA; 2 Department of Neurological Surgery, Northwestern University Feinberg School of Medicine, Chicago, IL USA

**Keywords:** Intrathecal baclofen, Baclofen withdrawal, Pump infection, Spasticity, Cerebral palsy, Pediatric neurosurgery

## Abstract

**Objective:**

Intrathecal baclofen (ITB) pumps are essential for managing spasticity and dystonia in children; however, they carry risks of hardware infection, withdrawal syndrome, and emergent failure. Management of these complications remains variable across institutions, and no unified, pediatric-specific workflow exists to date. We sought to develop and implement standard work tools (SWTs) to guide the evaluation and treatment of ITB pump infection and withdrawal in pediatric patients.

**Methods:**

Senior-level pediatric neurosurgery and physical medicine and rehabilitation (PM&R) physicians at a high-volume tertiary children’s hospital (Ann and Robert H. Lurie Children’s Hospital) collaboratively developed two structured SWTs addressing: (1) diagnosis and care of suspected ITB pump infection; and (2) structured weaning protocols to prevent and manage withdrawal during pump explantation or malfunction. SWTs were disseminated through detailed manuals and real-time clinical decision support. Their clinical utility was assessed through implementation in cases requiring pump interrogation or removal.

**Results:**

The SWTs were successfully applied across multidisciplinary teams; collectively, they standardize pump interrogation, laboratory evaluation, drug conversion strategies, ITB dose-based weaning thresholds, and escalation procedures for severe withdrawal or infection. The tools enabled consistent management of both emergent and subacute presentations. We further demonstrate their effectiveness through two representative cases: one involving MSSA pocket infection requiring pump removal and structured withdrawal management, and another involving non-inflammatory wound breakdown with preserved pump function requiring coordinated interdisciplinary care.

**Conclusions:**

SWTs improve safety and timeliness in the management of ITB pump infections and baclofen withdrawal in children. The presented tools provide a reproducible framework for first-line providers and pertinent specialists, particularly for those who may not be familiar with key signs and varied presentations. Broader adoption may reduce variability in treatment while optimizing longitudinal ITB therapy outcomes in pediatric patients.

## Introduction

Intrathecal baclofen (ITB) pumps are an FDA-approved therapy for spasticity and are commonly used off-label to treat dystonia in pediatric patients with cerebral palsy (CP) [1, 2]. Initially developed for adults with spasticity due to spinal cord injury and multiple sclerosis, ITB therapy was later shown to significantly reduce spasticity in children with CP [3, 4]. Subsequent studies have demonstrated that continuous ITB infusion also reduces dystonia in patients with CP [1, 5]. Compared to enteral baclofen, ITB offers greater bioavailability and fewer systemic side effects by delivering medication directly to the cerebrospinal fluid (CSF). However, this benefit comes with potential post-implantation complications, including infection, pump failure, and catheter malfunction [6, 7]. Reported hardware complication rates are estimated in some series to be 20–30% of patients with ITB pumps [8, 9]. Pump infection and removal can lead to subsequent withdrawal syndrome or sepsis if improperly managed, with reported infection rates ranging between 1 and 26% [10]. In children, withdrawal commonly presents within 12–48 h, initially as rebound spasticity, increased deep tendon reflexes, pruritus, irritability, or pain [11, 12]. If symptoms are allowed to persist in patients, they may progress to fever, autonomic instability, seizures, muscle rigidity, or multiorgan dysfunction [12, 13]. To aid in early recognition, a comprehensive list of critical signs, symptoms, and related treatment strategies is presented in Table 1, while Table 2 clearly defines decision thresholds for detecting implant infection and suggested next steps in management. The early identification of withdrawal is therefore critical to guide timely interventions and reduce the risk of serious sequelae.

**Table 1:** Clinical manifestations of baclofen withdrawal and suggested management. Symptoms of baclofen withdrawal are grouped by system with corresponding first-line interventions. This table serves as a rapid reference during ITB pump explantation in pediatric patients, especially in the context of infection. Management should be individualized based on symptom severity and patient status

System	Symptom	Intervention
Constitutional	Hyperthermia/Fever	If severe: dantrolene
	Pruritus	Cyproheptadine
Neurological	Hyperreflexia	Benzodiazepines, cyproheptadine, tizanidine, propofol, dexmedetomidine
	Tremor	
	Altered mental status	
	Seizure	
Cardiovascular	Autonomic Dysfunction (tachycardia, hypotension)	Monitoring and supportive care
Respiratory	Respiratory Failure	Noninvasive or invasive ventilation
Musculoskeletal	Hypertonia	Benzodiazepines, cyproheptadine, propofol If severe: dantrolene
	Rhabdomyolysis	IV hydration
Psychiatric	Hallucinations	Benzodiazepines, dexmedetomidine
	Delirium	

*ITB* intrathecal baclofen, *IV* intravenous

Additionally for all symptoms: ICU monitoring, restoring baclofen drug delivery (IT or enteral) [12, 13]

**Table 2:** Decision thresholds for suspected ITB pump infection. Clinical presentation, labs, and imaging-related thresholds for determining next steps, including further evaluation, intervention, or conservative management/observation

Domain	Parameter	Findings	Next steps
Clinical presentation	Fever	≥ 38.0 °C	Initiate sepsis evaluation; obtain labs and cultures
	Local erythema, warmth, tenderness	Present	Suspect pocket infection; avoid reservoir access
	Purulent drainage	Present	Culture drainage; consult neurosurgery and ID
	Clear wound drainage	Persistent drainage without erythema	Evaluate for CSF leak vs. wound breakdown
	Worsening spasticity without explanation	Present	Evaluate for withdrawal vs. infection
Laboratory markers	WBC	Rising trend	Supports systemic inflammatory response
	CRP	Rising trend	Concerning for infection
	ESR	Elevated above age-adjusted norm	Supportive but nonspecific
	Blood cultures	Positive	Confirm bacteremia; guide antibiotics
	CPK	Elevated	May suggest withdrawal-related hypertonicity
CSF evaluation	CSF WBC	Elevated above age-adjusted norm	May suggest meningitis
	CSF culture	Positive	Confirms CSF infection
	CSF glucose/protein	Low glucose/elevated protein	Concerning for infection
Imaging	Pump series X-ray	Hardware disruption	Consider malfunction
	Ultrasound (pocket)	Fluid collection	Consider aspiration
Reservoir/catheter access	Aspirate ≥ 1 cc CSF	Successful	Send for culture if infection suspected
	Unable to aspirate	< 1 cc CSF	Consider catheter obstruction; surgical evaluation
Disposition	Autonomic instability	Present	ICU admission
	Severe withdrawal	Present	ICU + sedation protocol
Operative threshold	Confirmed pocket infection (culture positive)	Present	Explantation recommended
	CSF infection	Present	Explantation + IV antibiotics
	Stable wound breakdown with negative labs	Present	Consider observation vs. revision

*C* Celsius, *CBC* complete blood count, *CMP* comprehensive metabolic panel, *CPK* creatine phosphokinase, *CRP* C-reactive protein, *CSF* cerebrospinal fluid, *ESR* erythrocyte sedimentation rate, *ICU* intensive care unit, *ID* infectious disease, *ITB* intrathecal baclofen, *IV* intravenous, *LP* lumbar puncture, *WBC* white blood cell count

The absence of standardized protocols for the management of ITB pump infections and the associated weaning process to prevent withdrawal syndrome highlights a critical gap in pediatric care. Standardized care protocols in pediatric neurosurgery have demonstrated significant benefits, including up to a 56% reduction in length of stay and a 43% decrease in direct cost for ventilator-dependent patients, as well as reduced infection rates for implanted hardware [14, 15]. Although ITB management protocols have been implemented in adult populations—with one group reporting a reduction in annual infection incidence of 3.1% [10]—there is a lack of comprehensive pediatric protocols for managing ITB pump infection, malfunction, or baclofen titration after explantation despite higher reported rates [16]. While isolated case reports exist, a standardized approach to care remains undeveloped. To address this gap, we present a tiered set of standard work tools (SWTs) developed at a high-volume pediatric neurosurgical center to support uniform, multidisciplinary management of ITB withdrawal following pump infection or removal.

## Methods

**Fig. 1:**
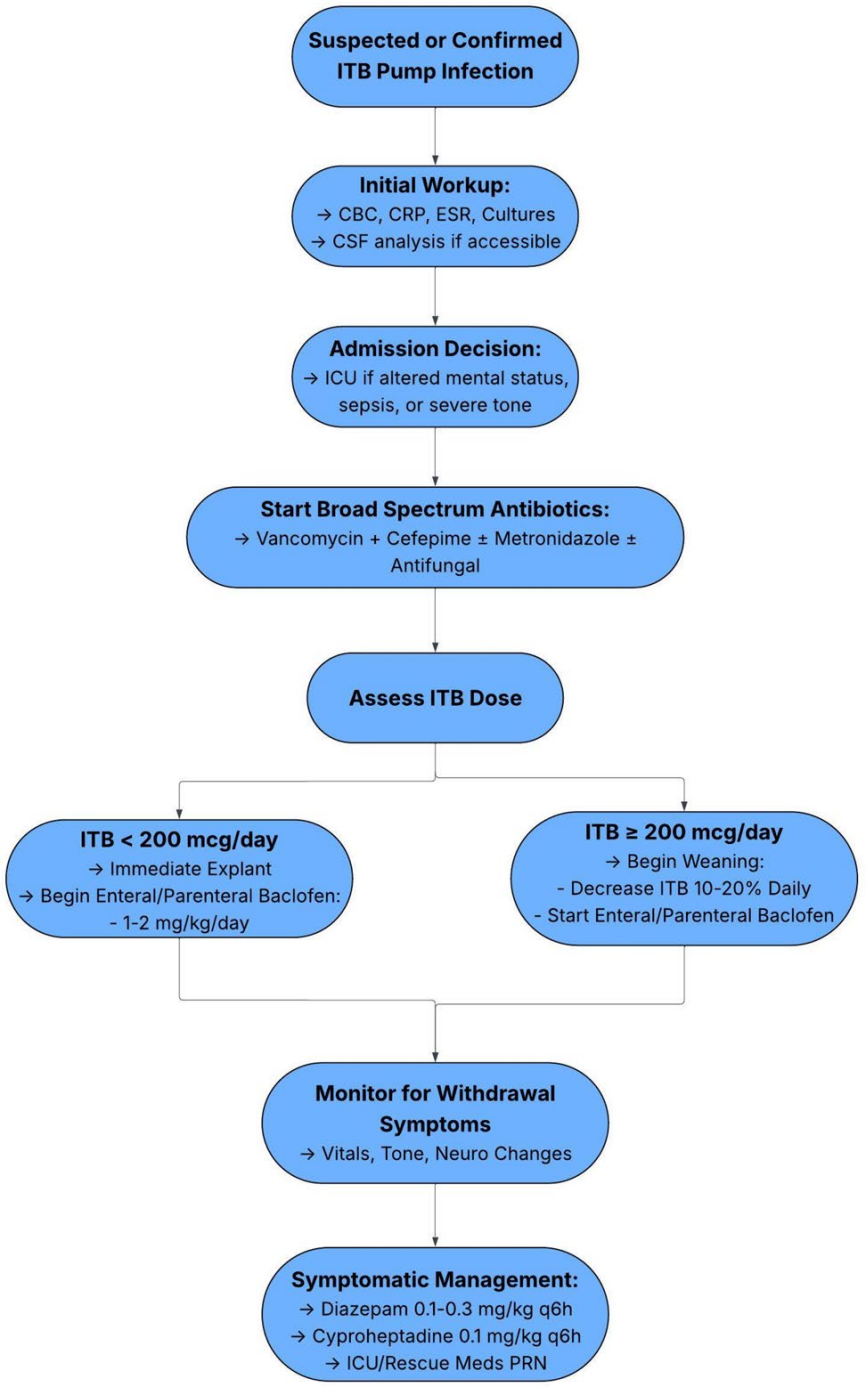
Algorithm for management of suspected or confirmed intrathecal baclofen (ITB) pump infection. Evaluation includes laboratory and CSF studies, empiric broad-spectrum antibiotics, and ICU admission for severe cases. Pump explantation and enteral baclofen conversion depend on ITB dose, with gradual weaning for higher doses. Patients are closely monitored for withdrawal, with symptomatic management as needed

SWTs for managing ITB withdrawal were developed over five years in response to recurring challenges with withdrawal following pump explantation due to infection in pediatric patients. A detailed, illustrated manual outlining the protocol, including recognition of early symptoms, bridging strategies, and escalation procedures, was distributed to the full multidisciplinary care team. This team includes pediatric neurosurgeons, neurologists, PM&R physicians, pediatricians, anesthesiologists, and perioperative nurses. The complete workflow is displayed in Fig. 1. The protocol is intended for children with underlying spasticity or dystonia managed with ITB therapy and builds on prior institutional experience with pump-related complications and withdrawal management, including previously published work by our group on outcomes following ITB pump removal [17]. We present a detailed overview of the workflow, as well as two illustrative cases in which the work tools were actively implemented to mitigate withdrawal following pump explantation.

### Pump interrogation and evaluation

When pump infection involving the pump pocket or intrathecal catheter is suspected or confirmed, the patient’s baclofen dose must first be evaluated. A patient receiving ≤ 200 mcg/day of ITB can generally be managed with escalating doses of antispasmodic medications such as enteral diazepam and baclofen, allowing for safe explantation of the pump and catheter system in the operating room (OR). Patients receiving daily doses exceeding 200 mcg are at risk for severe late-stage baclofen withdrawal, and a standard weaning protocol should often be followed. Of note, a threshold of 200 mcg/day is used as a risk-stratification guide and not a strict determinant of management. Patients receiving ≤ 200 mcg/day typically tolerate immediate explantation with structured enteral baclofen and benzodiazepine coverage. Those receiving > 200 mcg/day are at higher risk of delayed or severe withdrawal and therefore require anticipatory bridging therapy and higher-level monitoring. In patients receiving very high intrathecal baclofen doses, anticipatory weaning is often the immediate priority to prevent life-threatening tone escalation and autonomic instability, while infection evaluation and treatment should proceed concurrently whenever clinically feasible.

In either scenario, sepsis or baclofen withdrawal must also be actively managed while stabilizing the patient. Some emergencies require pump explantation and placement of a temporary intrathecal drain for continuous infusions. These patients should be continually monitored for signs of withdrawal (i.e., lower GABAergic activity) and include anxiety, pruritus, excessive spasticity, hypertonicity, tachycardia, hypertension, and fever (indicating pathologic muscle hypertonicity and resulting CPK-MB elevation). Early diagnosis is paramount in minimizing the complications of withdrawal syndrome and relies on multidisciplinary involvement. Withdrawal should be treated as a potentially life-threatening emergency.

### Admission

ICU admission is recommended for patients exhibiting signs of severe withdrawal, including autonomic instability or respiratory compromise, alongside intubation with barbiturate or propofol infusion as necessary. Patients with milder symptoms in the context of appropriate nursing and pharmacy resources may be monitored on the hospital ward with support from the pediatric hospitalist service. After consultation with neurosurgery, PM&R, infectious disease (ID), and pharmacy teams, initial laboratory testing should include (but is not limited to): C-reactive protein/erythrocyte sedimentation rate (CRP/ESR), complete blood count (CBC) with differential, blood cultures, creatine phosphokinase (CPK), comprehensive metabolic panel (CMP), and coagulation studies. Disposition will depend on institutional culture and resources.

If a pocket infection is suspected, port access should be avoided. Interventions may include pump interrogation, insertion of a large bore IV as necessary, consideration of a peripherally inserted central catheter (PICC) with negative blood cultures, chest X-ray, potential Foley catheter insertion for urine monitoring, and continuous monitoring with pulse oximetry and EKG. If CSF infection is suspected, cerebrospinal fluid sampling should be performed either via reservoir access or lumbar puncture depending on safety and catheter patency. Reservoir tapping is preferred when the system is intact and accessible; however, lumbar puncture is recommended when catheter obstruction is suspected, when access port tapping carries a high risk of contamination, or when meningitis or ventriculitis is a concern.

### Medication management

Pharmacologic management is central to both inpatient and outpatient treatment of baclofen withdrawal and pump-related infection. While we suggest general ranges for pharmacomanagement, the treating clinicians should defer to personal judgment regarding drug-drug interactions and the patient’s physiological state to avoid oversedation and undertreatment. Management largely consists of core pharmacologic agents including enteral baclofen (10–25 mg qid, GABA-B agonist) and diazepam (1–8 mg q8h; GABA-A agonist), with adjuncts based on symptom severity. Additional medications may be used based on symptom severity, including rescue IV lorazepam (1–4 mg q6h), enteral tizanidine (alpha-2 adrenergic agonist), cyproheptadine (first generation anti-histamine; 4–8 mg q6-8 h), enteral dantrolene (muscle relaxant; 25–100 mg daily), and enteral gabapentin (pain management). Alternative strategies include bolus delivery via the ITB pump during tapering or a temporary pump to maintain intrathecal access [18, 19]. Avoid the use of opiates for pain control due to significant risk of respiratory failure when combined with baclofen and Valium (diazepam). Suspected pump infection without an organism should be started on appropriately weight-based doses of broad-spectrum antibiotics (e.g., vancomycin, cefepime, metronidazole ± antifungal coverage). When a pump is explanted, especially if recently implanted, the patient’s pre-implant enteral regimen (e.g., baclofen, diazepam, tizanidine, gabapentin) should be reviewed and reinstated as appropriate. Systemic infection and physiologic stress commonly worsen spasticity and can increase medication requirements [20]. Accordingly, dosing should be dynamically titrated based on clinical response rather than strict conversion ratios; this is particularly important considering the confounding effects of physiologic stressors, namely systemic infection, on neuromuscular allostasis.

### ITB weaning

**Fig. 2:**
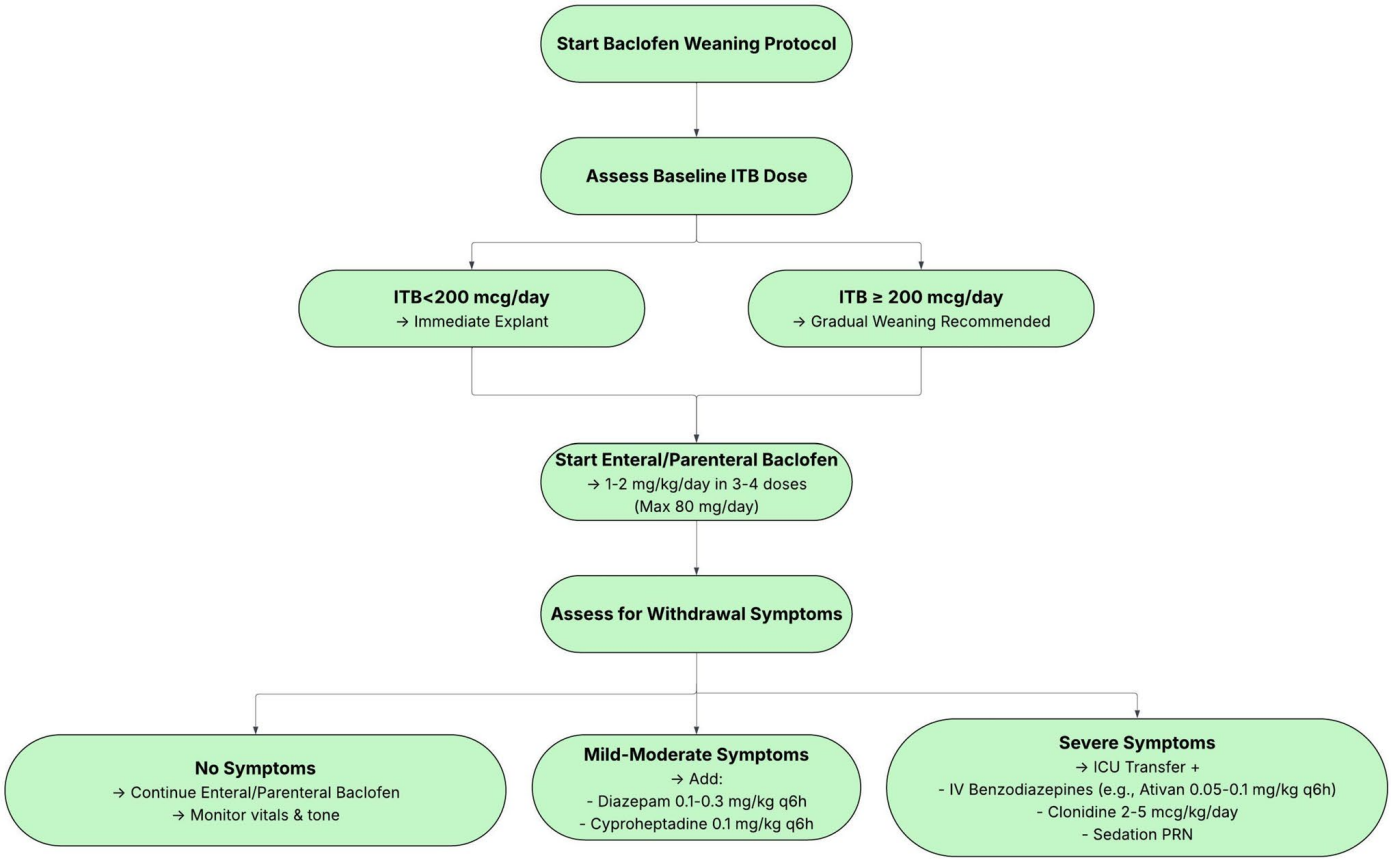
Baclofen weaning protocol following intrathecal baclofen (ITB) pump explantation. Management is guided by baseline ITB dose, with immediate explantation for doses < 200 mcg/day and gradual weaning for ≥ 200 mcg/day. Enteral baclofen is initiated at 1–2 mg/kg/day, and patients are monitored for withdrawal symptoms. Supportive management includes benzodiazepines, cyproheptadine, or clonidine as indicated, with ICU transfer for severe cases

In non-emergent cases the goal for ITB weaning criteria should be met within seven days (see Fig. 2). Regardless of urgency, physicians should correlate the weaning protocol with patient-specific physiology. ITB dose can typically be reduced by 80–200 mcg daily with increasing doses of enteral medications and intravenous dexmedetomidine and propofol as necessary. Acute ITB withdrawal due to a failed pump or catheter dysfunction can be evaluated by a CT myelogram. Acute withdrawal from a primary pump or catheter system malfunction should be urgently addressed surgically.

### Catheter access protocol

If the pump is known to be uninfected, but the cause of aberrant functionality is still unknown, CSF may be aspirated through the catheter access port for downstream culturing and tests. Using a 10-cc syringe and a 24-gauge non-coring needle, CSF should be aspirated from the iodine-prepped access port, after which the needle is removed and discarded appropriately. A catheter bolus should then be programmed to prevent iatrogenic withdrawal. If at least 1 cc CSF is not able to be aspirated (roughly 3X the volume of a completely intact catheter system), the catheter must be considered blocked and no attempt to clear the catheter should be made. This catheter dysfunction should be surgically addressed.

## Results

The ITB pump infection and withdrawal management SWTs have been in use at a large free-standing children’s hospital for the last three years. We present two illustrative cases outlining their robust and nuanced implementation.

### Illustrative cases

#### Case 1

A 6-year-old male was transferred to the Lurie Children’s Hospital Emergency Department (ED) (day 0, D0) for evaluation of a fever and edema around his ITB pump on POD 21 from primary implant. Initial laboratory tests were notable for a WBC count of 12.1 × 10^9^/L, and a CRP of 5.6 mg/dL, which responded to a single dose of ceftriaxone.

On D2, the patient underwent a 7 mL aspiration of the fluid pocket surrounding the ITB pump which grew methicillin sensitive *S. aureus* (MSSA). Infectious disease consultation recommended IV doxycycline. The ITB dose was 120 mcg/day and was determined to be safe to remove as it was below the threshold requiring weaning (200 mcg/day); all medications for the patient were further reviewed. Following explantation of the pump and hardware, he was transferred to the PICU, with dystonia and agitation managed via IV dexmedetomidine. Worsening dystonia prompted IV diazepam 3.5 mg q6h and an increase in dexmedetomidine. Doxycycline was converted to IV cefazolin due to the absence of meningitis. Enteral baclofen (2.5 mg tid) and cyproheptadine (2 mg q8h) were initiated.

By D5, the patient showed clinical improvement in baclofen withdrawal symptoms. The patient remained on scheduled enteral baclofen therapy (tid), cyproheptadine, and diazepam (3.5 mg q6h) while being weaned off dexmedetomidine as the baseline clonidine dosage was increased. On D6, the patient displayed manageable dystonia. Enteral baclofen was continued, dexmedetomidine and cyproheptadine were discontinued, and the diazepam dosage was reduced. On D7, the patient was transferred to the ward. IV oxacillin was initiated for a 2-week course to cover the MSSA. PO baclofen was maintained. The patient’s labs improved commensurate with his clinical syndrome (WBC count of 7.13 × 10^9^/L and a CRP of < 0.3 mg/dL), and he discharged home on D8.

#### Case 2

A 26-year-old male with a history of traumatic brain injury (TBI) presented with clear fluid drainage from the lumbar incision, 44 days after a surgery that revised both the abdominal pump and the original intrathecal catheter. Prior to ED presentation, the patient had noted discharge from the lumbar wound as early as four days postoperatively; the possibility of a CSF leak was ruled out following review of the patient’s operative course. The patient was prescribed 300 mg clindamycin for 7 days on postoperative D28. Follow-up with the patient on postoperative D36 revealed a closed, well-appearing incision.

In the ED, the surgical incision appeared clean and dry, with absent tenderness, warmth, and erythema. However, the caregivers reported a clear serous discharge from the distal portion of the incision, and it was reproduced on a physical exam. Initial labs displayed an ESR of 4 mm/hr, neutrophils of 46.7%, and a WBC of 5.26 × 10^9^ cells/L. The patient was admitted for observation, and x-ray imaging showed expected course and connectivity within the limits of visibility. The patient was prescribed cefepime (2000 mg) and vancomycin (1000 mg q8h). Neurosurgical consultation determined non-inflammatory wound breakdown and non-healing surgical site. Given the patient’s baclofen dose of 650 mcg/day and the family’s desire to avoid a prolonged hospitalization or period without the pump, the patient instead had a new baclofen pump placed within the left abdomen and a new catheter system positioned through an incision above the open back wound. Along with complete explantation, the patient underwent wound washout, complex closure of the open draining back wound, and removal of the right abdominal pump. Intraoperative sampling of lumbar wound drainage subsequently grew *Pseudomonas aeruginosa*, confirming wound contamination. Although delayed reimplantation following infection clearance remains recommended practice in the presented protocol, immediate explantation with simultaneous reimplantation has been reported as a safe alternative in carefully selected patients to mitigate withdrawal risk and prolonged interruption of therapy [21]. The patient was chosen as an exception given clear CSF and tone requiring immediate management.

ID subsequently revised the antibiotics course to levofloxacin 750 mg daily for 6 months. All other pre-explantation medications were reviewed and determined suitable to continue. On D6 (post-operative D50), the patient’s ESR was 24 mm/hr, CRP 2.5 mg/dL, and WBC count 5.66 × 10^9^/L. On D8, the patient remained afebrile, with an ESR of 21 mm/hr, CRP of 0.9 mg/dL, and a WBC count of 5.4 × 10^9^/L. There was no disruption in ITB dosing throughout the patient’s admission, so there was no alteration of medical management for spasticity or dystonia. The patient discharged home and followed up with neurosurgery via outpatient therapy, off antibiotics at 6 months and without further need at one year.

## Discussion

The cases presented demonstrate the effectiveness of a multidisciplinary approach in managing ITB withdrawal secondary to pump infection. The care of patients undergoing withdrawal due to the removal of intrathecal drug delivery systems (IDDS) is poorly standardized, given the limited number of patients requiring such device-mediated interventions. Although IDDS have an overall favorable safety profile and well-established clinical benefit, infections remain a significant concern, especially in pediatric and high-risk populations. Prompt and aggressive treatment of ITB pump infections is essential to reduce the risk of morbidity associated with pump explantation and to preserve long-term options for spasticity management. A retrospective study of 341 pediatric cerebral palsy patients with ITB pumps displayed an infection rate of 6.9% per procedure, with 14.6% of all patients having an infection. Early infection was associated with CSF leakage, which led to complications such as headaches, prolonged medical management, and, in some cases, the need for procedural repair [17, 22].

Varied approaches have been implemented to manage IDDS infections, including local and intra-reservoir administration of antibiotics [23–25], parenteral antibiotics and response assessment prior to explantation [26, 27], and medical management in conjunction with immediate explantation. Standardized work tools have become increasingly beneficial in formulating hospital protocol for the management of implantable drug delivery systems, particularly with respect to preventing infection during IDDS insertion [17, 28]. Opportunities to provide education to both practicing neurosurgeons and other members of the interdisciplinary care team have proven highly effective in reducing complications and instituting standard of care protocols, as evidenced by the establishment of the NANS educational curriculum for IDDS implantation and patient management [29].

The illustrative patient cases highlight adherence to standard workflows developed by our interdisciplinary team for managing baclofen weaning. Case 1 demonstrates the prompt evaluation of a patient presenting with evidence of infection, coordination with the infectious disease team, successful pump explantation, and management of postoperative baclofen wean. The clinical decision tool provided a framework for complex physiological care. Broad-spectrum antibiotics were promptly initiated, and the pump was rapidly explanted per protocol, as the patient was receiving < 200 mcg/day of ITB. The second case demonstrated protocol use in a patient displaying ITB pump infection with immediate implantation of a new ITB pump. The neurosurgical and infectious disease teams coordinated to promptly manage infection while maintaining muscle tone therapy.

There are several limitations to this study. This study focuses on developing standardized workflows for managing ITB complications such as infection and baclofen weaning after pump removal. Long-term outcomes were not reported. Due to the limited sample size, our proposed protocols for baclofen weaning may not represent the comprehensive range of baclofen withdrawal presentations but serve as a framework for clinical intervention. Implementation depends on close coordination across multiple specialties which are not always present across institutions. Deviations from the protocol may also occur on a patient-specific basis and in the context of their clinical presentation. Given the variety of presentations and heterogeneity among pediatric patients with conditions eliciting spasticity, the needs of individual patients may fundamentally differ from the care outlined in these SWTs. These may include children with several comorbidities, multiorgan failure, or treatment-refractory epilepsy. Medical dose recommendations are guides only and should be contextualized by the clinical providers’ medical knowledge.

## Conclusions

ITB pump infection and malfunction are rare enough occurrences that clinicians generally do not have standard routine algorithms for effective treatment. These SWTs may serve as a reliable framework for multidisciplinary care of these patients in acute settings. We encourage widespread adoption of SWTs to manage ITB pump infections and promote further investigation into optimal weaning protocols for patients receiving higher ITB dosages.

## Data Availability

No datasets were generated or analysed during the current study.
